# Effectiveness of a New Exercise Program after Lower Limb Arterial Blood Flow Surgery in Patients with Peripheral Arterial Disease: A Randomized Clinical Trial

**DOI:** 10.3390/ijerph110807961

**Published:** 2014-08-07

**Authors:** Edita Jakubsevičienė, Donatas Vasiliauskas, Linas Velička, Raimondas Kubilius, Eglė Milinavičienė, Jonė Venclovienė

**Affiliations:** 1 Institute of Cardiology, Medical Academy, Lithuanian University of Health Sciences, Sukilėlių St. 17, Kaunas 50161, Lithuania; E-Mails: donatas.vasiliauskas@lsmuni.lt (D.V.); j.vencloviene@gmf.vdu.lt (J.V.); 2 Department of Cardiothoracic and Vascular Surgery, Lithuanian University of Health Sciences, Eivenių St. 2, Kaunas 50009, Lithuania; E-Mail: lvelicka@gmail.com; 3 Department of Cardiology, Medical Academy, Lithuanian University of Health Sciences, Eivenių St. 2, Kaunas 50009, Lithuania; E-Mail: raimondas@efarmacija.lt; 4 Viršužiglis Hospital of Rehabilitation, Hospital of Lithuanian University of Health Sciences, Viršužiglis 53193, Lithuania; E-Mail: egle.milinaviciene@kaunoklinikos.lt; 5 Department of Rehabilitation, Medical Academy, Lithuanian University of Health Sciences, Eivenių St. 2, Kaunas 50009, Lithuania; 6 Department of Environmental Sciences, Vytautas Magnus University, Vileikos St. 8, Kaunas 44404, Lithuania

**Keywords:** physical therapy program, lower-limb bypass surgery, peripheral arterial disease

## Abstract

*Objective*: The aim of this study was to evaluate the effectiveness of a supervised exercise program (SEP) plus at home nonsupervised exercise therapy (non-SET) on functional status, quality of life (QoL) and hemodynamic response in post-lower-limb bypass surgery patients. *Results*: One hundred and seventeen patients were randomized to an intervention (n = 57) or a control group (n = 60). A new individual SEP was designed for patients with peripheral arterial disease (PAD) and applied to the studied subjects of the intervention group who also continued non-SET at home, whereas those assigned to the control group received just usual SEP according to a common cardiovascular program. The participants of the study were assessed by a 6-min walking test (6 MWT), an ankle-brachial index (ABI), and the Medical Outcomes Study Short Form-36 (SF-36) of QoL at baseline, at 1 and 6 months after surgery. A significant improvement was observed in the walked distance in the intervention group after 6 months compared with the control group (*p* < 0.001). The intervention group had significantly higher QoL score in the physical and mental component of SF-36 (*p* < 0.05). *Conclusions*: A 6-month application of the new SEP and non-SET at home has yielded significantly better results in walking distance and QoL in the intervention group than in the controls.

## 1. Introduction

Peripheral arterial disease (PAD) is one of the complications of atheroslerosis, which leads to arterial obstruction and decreased blood flow to the lower extremities. The risk of developing PAD can be predicted by the patient’s age and well-defined atherosclerotic risk factors, including cigarette smoking, hyperlipidemia, diabetes mellitus, and arterial hypertension. The prevalence of PAD increases with aging; in the general population it accounts for 12%–14% affecting up to 20% of subjects over 75 years [1]. The most frequent clinical symptomatic manifestation of PAD is intermittent claudication (IC), which is defined as a reproducible lower extremity muscular pain induced by exercise and relieved by rest. Since the superficial femoral and popliteal arteries are most commonly affected by atherosclerosis [2], the pain of intermittent claudication is frequently confined to the calf. However, more than a half of all people with PAD are asymptomatic or have atypical symptoms [3]. PAD is strongly associated with cardiovascular disease. Many people diagnosed with PAD are at high risk of further cardiovascular events [3]. It is associated with increased mortality and significant morbidity including major limitations in mobility and physical functioning, and decreased quality of life [1].

The treatment strategies of PAD comprise lifestyle changes such as smoking cessation, exercise and medication. Patients with severe symptoms that are inadequately managed are frequently referred to secondary care institutions to assess endovascular testing, surgical revascularization, and amputation. Bypass surgery is used for the treatment of severe lifestyle-limiting disability when angioplasty has been unsuccessful or is unsuitable, and imaging has confirmed that bypass surgery is appropriate for the individual [4,5,6].

Exercise rehabilitation programs including secondary prevention may benefit patients with PAD by preserving or improving functional capacity and reducing cardiovascular events [3]. Secondary prevention programs integrate exercise into the overall treatment plan that includes lipid management, blood pressure control, smoking cessation, nutrition education, body weight reduction, and diabetes mellitus treatment. The goal of such programs is to reduce physical disability and cardiovascular risk by restoring optimal physical, psychological and social functioning [3,7]. One of the main health care goals in PAD after lower-limb bypass surgery is to integrate the use of long-term rehabilitation programs to provide as much as possible longer treatment regimen without interruption. The basic component of the rehabilitation is supervised exercise therapy (SET). SET is an effective tool in the treatment of claudication and is currently recommended as a first-line therapy for these patients (a Class IA level of evidence) [8]. Non-supervised exercise therapy (non-SET) is indicated when SET is not feasible or available (a Class IC level of evidence) [5]. Patients who are unable or unwilling to participate in SET may choose home-based walking exercise intervention, which is effective in patients with PAD [9,10].

However, there is still a lack of studies showing the best SET method and proving the effectiveness of rehabilitation after lower-limb arterial blood flow surgery. Also, the effectiveness of post-operative SET plus non-SET has not been evaluated. Moreover, little is known about the effects of exercise therapy, particularly for longer-term follow-up after lower-limb bypass surgery. However, current guidelines suggest that pharmacotherapy, supervised exercise rehabilitation and lower extremity bypass surgery are effective therapies for patients with PAD [7]. Patients who underwent lower extremity bypass grafting surgery showed significant improvements in self-reported physical functioning, walking distance, bodily pain, ankle-brachial index (ABI), and leg symptoms [11]. However, the effects of exercise combined with surgery are not widely described. To our knowledge, only two studies analyzed the effect of exercise therapy after lower-limb arterial blood flow surgery [12,13]. The purpose of this randomized study was to assess the effectiveness of the new supervised exercise program (SEP) and non-SET at home for 6 months following lower limb bypass surgery on physical function, limb hemodynamics, and quality of life (QoL).

## 2. Methods

### 2.1. Study Design

The study included 162 patients hospitalized in the Department of Cardiothoracic and Vascular Surgery, Hospital of the Lithuanian University of Health Sciences (LUHS) between the period of June 2012 and March 2014, who were diagnosed with the superficial femoral artery occlusion and underwent femoral-popliteal artery bypass grafting.

Subject inclusion criteria consisted of patients with stage II–III of PAD as defined by Fontaine who underwent femoral-popliteal artery bypass grafting surgery and gave permission to participate in the clinical study as well as to continue treatment in rehabilitation centers.

Subjects were excluded if they had Fontaine stage IV (PAD associated with tissue loss) and had undergone limb amputation, did not give consent to participate in the study, had other diseases, which reduced walking ability (*i.e.*, orthopaedic problems, spinal stenosis, angina pectoris or dyspnoea), refused to go to rehabilitation centers or were subjected to another artery segment operation (*i.e.*, aorto-iliac, infra-genicular).

### 2.2. Ethical Considerations

All subjects gave their informed consent for inclusion before they participated in the study. The study was conducted in accordance with the Declaration of Helsinki, and the protocal was approved be the Kaunas Region Biomedical Research Ethics Committee of LUHS (No. BE-2-22).

### 2.3. Randomization

Patients were randomized into two groups, an intervention (n = 81) and a control (n = 81), according to a random sampling approach 1:1. Individuals in the control group received rehabilitation with usual SEP followed in rehabilitation centers, where the general program of cardiovascular diseases (CVD) was applied, whereas those assigned to the intervention group continued treatment in rehabilitation centers, according to the new SEP developed for PAD patients and completed non-SET at home for 5 months, according to the individualized programs arranged by a physical therapist. Both groups received optimal pharmacological treatment [5].

### 2.4. Post-Operative Care

Subjects’ hospitalization after the operation took an average of 7 days of treatment, then early rehabilitation and optimal medical treatment were started. Clinical Cardiology Laboratory at the Institute of Cardiology in LUHS has prepared a methodology of physical therapy for this group of patients. During the first 2 days, static breathing exercises, relaxation, and general light small muscle training exercises were used; in the time of the next 5 days, dynamic breathing exercises, physical exercises for medium size and large muscle developing, and walking therapy were applied. The intervention and control groups were referred from the hospital to rehabilitation centers where treatment lasted for 18 days. The studied rehabilitees were referred to Viršužiglis Hospital of Rehabilitation, “Dainava” or “Draugystė” sanatoria. The rehabilitation program consisted of pharmacologic treatment, evaluation of risk factors and their modification, SET (twice a day), occupational therapy, physiotherapy, therapeutic massage, psychologist and social worker consultations. The rehabilitation program was carried out six times a week. The head of rehabilitation center and physical therapists were involved in the investigation and took care of the studied. Rehabilitation program was designed for every patient individually in accordance with the patient’s needs.

### 2.5. Control Group

SET for the control group in rehabilitation centers was applied following the general program of CVD in groups and individually. The general program consisted of combined aerobic and resistance training. It was performed using the treadmills, ergometers, steppers, stair climbing and jogging and resistance devices in the rehabilitation centers under supervision of a physical therapist. Each session lasted up to 60 min, including a 10-min warm-up followed by 40 min of aerobic and resistance training, and finally, a 10-min cool-down. The intensity of training was based on clinical conditions and established between 60%–85% of maximum heart rate. After 18 days of rehabilitation treatment, the subjects in the control group were recommended to continue physical training programs and the optimal pharmacological treatment (under supervision of a family doctor).

### 2.6. Intervention Group

The studied rehabilitation centers have introduced a new SEP for the intervention group according to the recommendations [8,14]. Individual physical therapists worked with the subjects up to 45 min. Individually, procedures consisted of the following: (1) a 5–10 min warm-up consisting of dynamic breathing and stretching exercises; (2) lower limb exercising including track walking, stair climbing, treadmill exercise; (3) a 5–10 min cool-down consisting of static and dynamic breathing and stretching exercises. The intensity of training was based on clinical conditions and established between 60%–85% of maximum heart rate. After 18 days of rehabilitation, the intervention group was assigned to an individualized physical activity and continued optimal pharmacological treatment at home for five months (under supervision of a family doctor). Non-SET was based on recommendations of a physical therapist. In this program, patients were asked to walk for at least 30 min a day, three to five times a week, and to increase their walking time as often as possible, including a warm-up phase and ending with a cool-down period. In addition, the studied rehabilitees were provided with a physical activity diary that included instruction on how to complete it. In the diary, the studied subjects had to indicate the date, description and duration of activity as well as their wellbeing.

### 2.7. Measurements

Both groups were assessed according to the following measurements before the surgery. Patients’ body weight and height were measured in accordance with guidelines of the International Society for the Advancement of Kinanthropometry [15]. Patient blood tests made before surgery were obtained from a family doctor and hyperlipidemia was estimated. Diabetes mellitus was defined in accordance with the case history of the patient. Hypertension was considered if systolic blood pressure was of 140 mm Hg or higher, diastolic blood pressure of 90 mm Hg or higher, or previously diagnosed and treated high blood pressure [16]. Cigarette smoking was based on history of current smokers if they smoked within the last month. History of CVD (myocardial infarction, angina, or stroke) was considered when it was diagnosed by a physician.

A six-min walk test (6 MWT). The 6 MWT is a submaximal test showing the distance which the patient can quickly walk on a flat, hard surface in a period of 6 min. The test was symptom-limited, so the patients who became symptomatic (e.g., with lower extremity muscular pain, severe dyspnea, dizziness) were told to stop walking and restart when possible. The distance walked up to the onset of pain (pain-free walking—PFW) during the 6 MWT and total distance walked during the test were recorded. The survey was conducted according to a standard protocol [17], in the hallway with marked walking distance and walking time measured with a stop watch. The 6 MWT reflects the patient's level of physical fitness in daily life [18]. Before and after the test, resting heart rate and blood pressure of patients were measured and recorded. This test is frequently used because it is standardized and validated in the functional evaluation of the chronic, disabling diseases [19]. The test was performed preoperatively, at 1 month and 6 months later after the surgery.

Ankle-brachial index (ABI) defined as a ratio of the ankle and brachial artery systolic pressure, was used as one of the most common methods for diagnosis of PAD [20]. Measurement of the ABI is indicated as a first-line noninvasive test for screening and diagnosing lower extremity artery disease (Class I, Level B) [5]. ABI measurement was made on the same limb artery before and at 1 and 6 months later after the surgery. A handheld Doppler probe (Multi Dopplex II; Huntleigh Health Care, city, state abbrev if US, country) was used to obtain systolic blood pressure mean in the dorsalis pedis and posterior tibial arteries according to the recommendations [20].

Quality of life (QoL). Health-related QoL was assessed using the Medical Outcomes Study Short Form 36 (MOS SF-36) General Health Survey [21]. It is one of the most commonly used questionnaires, which is applicable for different health problems. The SF-36 is recommended as one of the most appropriate generic instruments for this patient group [22]. It consists of 36 questions that represent the eight areas of life which are connected with the two main dimensions of health: physical and mental health. Physical health reflects physical functioning, role limitations due to physical problems, bodily pain, general health perception, whereas mental health is defined by vitality, social functioning, role limitations due to emotional problems, emotional state. For each subscale, item scores were recorded, summed, and included into a scale from 0 to 100, with better health states resulting in higher score. QoL questionnaire SF-36 was completed before and at 6 months after the surgery.

### 2.8. Statistical Analysis

The study data statistical analysis was performed using the SPSS for Windows 20.0 program. Quantitative indicators of the rehabilitation and control groups were compared using Student’s (t) test, the mean value standard errors (±SE) were presented; qualitative—using Chi-square (x2) test. The treatment effect after 1 and 6 months was assessed using analysis of covariance (ANCOVA). In the ANCOVA, the data after 1 and 6 months were used as dependent variables, the variables before the operation—as a covariate, and the patients group—as a factor. Verification of statistical hypotheses selected statistical confidence level of *p* < 0.05 (95% statistical confidence).

## 3. Results

**Figure 1:**
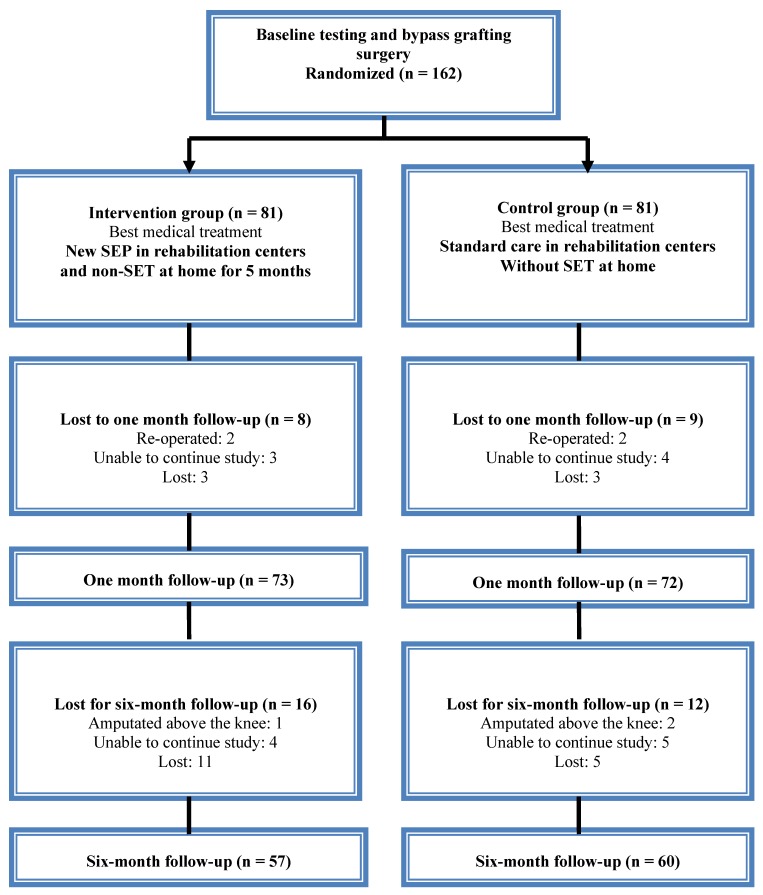
CONSORT flow chart of the study.

Out of 162 studied patients, 45 failed to complete the full study follow-up period. Of those, four patients were re-operated, three patients had amputation above the knee, sixteen patients were unable to continue study due to health problems, and twenty-two patients were lost for follow-up examination (Figure 1).

Given demographical characteristics (age, gender), atherosclerosis-inducing factors (BMI, hyperlipidemia, diabetes mellitus, hypertension, and smoking) and cardiovascular disease, no significant differences (*p* > 0.05) were observed in the intervention and control groups before surgery. The general participant characteristics are shown in Table 1.

**Table 1:** Clinical characteristics of the intervention (n = 57) and control groups (n = 60).

Variable	Intervention Group	Control Group	*p*
Age (± SE)	67.4 ± 1.0	66.5 ± 1.0	0.535
Gender (men) (%)	52 (91.2)	57 (95)	0.419
BMI, kg/m^2^ (± SE)	26.8 ± 0.6	27.2 ± 0.5	0.630
Hyperlipidemia (%)	14 (24.6)	11 (18.3)	0.411
Diabetes mellitus (%)	5 (8.8)	3 (5)	0.419
Hypertension (%)	40 (70.2)	45 (75)	0.558
Current smoker (%)	47 (82.5)	41 (68.3)	0.077
Cardiovascular disease (%)	12 (21.1)	10 (16.7)	0.544

Notes: BMI = body mass index; ±SE = standard error.

### 3.1. Physical Function

**Figure 2:**
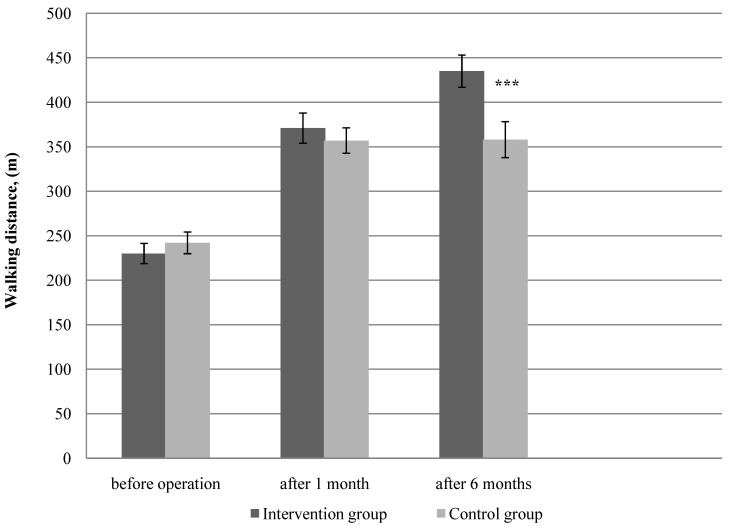
The total six-min walk test measurements at baseline, at 1 and 6 months after the surgery in the intervention (n = 57) and control groups (n = 60).

Notes: Error bars indicate ± standard error; m = meters; months = after bypass surgery; *** *p* < 0.001.

**Figure 3:**
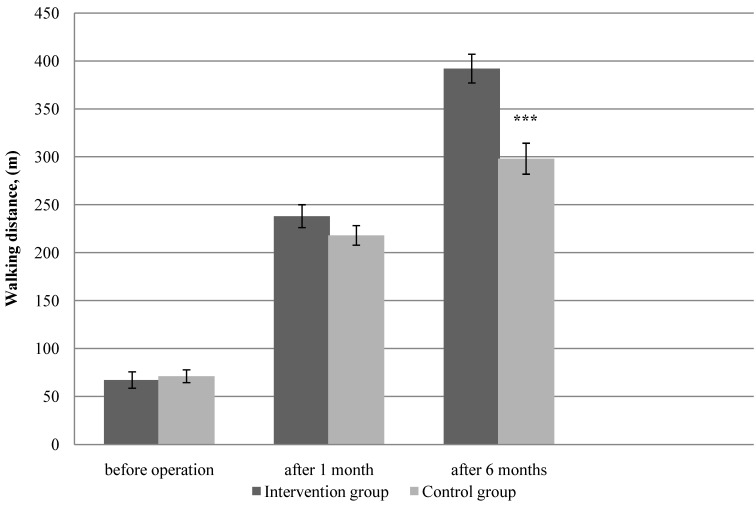
Pain-free walking distance measurements during six-min walk test at baseline, at 1 and 6 months after the surgery in the intervention (n = 57) and control groups (n = 60).

Notes: Error bars indicate ± standard error; m = meters; months = after bypass surgery; *** *p* < 0.001.

Physical function measured by total 6 MWT and PFW during 6 MWT, showed a significant difference between the two groups at the 6-month follow-up (Figure 2—*p* < 0.001, Figure 3—*p* < 0.001, respectively). Assessment of physical function at baseline did not show differences in mean total 6 MWT and PFW distance between groups (*p* > 0.05). In the PFW during 6 MWT and the total 6 MWT both groups achieved a statistically significant increase in walking distance at one month follow-up; however, there was obtained no significant difference between the studied groups. Already 6 months later after the surgery, a significant improvement was observed in PFW during 6 MWT and the total 6 MWT in the intervention group compared with the control group. The intervention group showed a greater change in walking distance (PFW, 6 MWT) than the control group. When analyzed as percentage mean change from baseline to 6 months, the mean change in the total 6 MWT was 89% in the intervention group and 47% in the control group, respectively. For the PFW during 6 MWT, the percentage mean increase from baseline was 485% in the intervention group, and 319% in the control group.

### 3.2. Limb Hemodynamics

**Figure 4:**
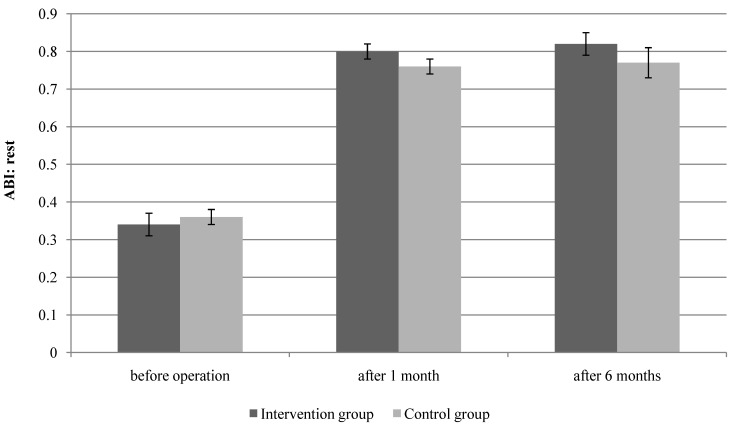
Peripheral hemodynamic measurements at baseline, at 1 and 6 months after the surgery in the intervention (n = 57) and control groups (n = 60). ABI = ankle-brachial index; error bars indicate ± standard error; months = after bypass surgery.

Assessment of the lower extremity circulation before the surgery did not show statistically significant difference between the groups: ABI in the intervention group was 0.34 ± 0.03; in the control group 0.36 ± 0.02, respectively. Analysis of ABI means did not reveal statistically significant difference between the groups after one and six months which was expected due to the nature of surgical treatment (*p* = 0.071 and *p* = 0.063, respectively) (Figure 4).

### 3.3. SF-36 of QoL

QoL measured using SF-36 showed a statistically significant difference between the two groups during the 6 months of follow-up in the domains of physical function (*p* = 0.001), bodily pain (*p* < 0.001), vitality (*p* = 0.002), and social function (*p* = 0.048). The same was true to the SF-36 physical component score (*p* < 0.001) and mental component score (*p* = 0.020) (Table 2). The remaining domains showed no statistically significant difference between the groups during the follow-up.

**Table 2:** Health-related quality of life at baseline and at 6 months after the surgery in the intervention (n = 57) and control groups (n = 60).

Quality of Life	Before Operation			After 6 Months		
	Intervention Group	Control Group	*p*	Intervention Group	Control Group	*p*
Physical health score	31.88 ± 2.96	31.97 ± 2.03	0.939	57.73 ± 2.56	47.75 ± 2.74	0.000
Physical function	31.22 ± 1.58	30.91 ± 2.70	0.679	62.28 ± 2.61	49.23 ± 3.90	0.000
Role-physical	30.26 ± 3.19	32.91 ± 2.33	0.406	51.05 ± 4.47	42.37 ± 4.57	0.182
Bodily pain	29.04 ± 2.03	27.40 ± 1.76	0.543	65.69 ± 2.49	54.23 ± 4.89	0.000
General health	37.01 ± 1.50	35.75 ± 1.32	0.527	47.54 ± 2.65	45.25 ± 2.74	0.357
Mental health score	52.17 ± 2.68	49.72 ± 1.63	0.300	64.35 ± 3.57	59.11 ± 3.37	0.020
Vitality	42.01 ± 4.25	39.73 ± 2.20	0.302	59.38 ± 2.39	48.30 ± 4.25	0.002
Social function	40.35 ± 3.99	38.33 ± 2.72	0.446	68.61 ± 4.34	61.66 ± 4.46	0.048
Role-emotional	60.05 ± 8.45	61.77 ± 7.80	0.876	67.73 ± 6.50	65.65 ± 5.14	0.899
Emotional state	59.64 ± 4.85	55.46 ± 2.50	0.091	67.43 ± 3.79	63.20 ± 2.76	0.218

Note: Months = after bypass surgery.

## 4. Discussion

This is the first investigation which assessed the effectiveness of a new SEP program plus non-SET at home for 5 months according to the program arranged by a physical therapist in patients after a lower-limb bypass surgery.

The major findings of this investigation were as follows: significantly longer walking distance and significantly better score in the physical and mental health components of QoL in the intervention group than in the control group during a six-month study. However, ABI results were not significantly different between the two groups during the 6 months of follow-up, although the results of ABI have shown a trend towards better results in the intervention group compared with the control group.

A statistically significant improvement was found in walking distance and ABI after one month follow-up for both groups, however, there was no statistically significant difference between the groups. It might be reasonable to think that both groups were strongly influenced by the surgery. The hypothesized effects of the new SEP after lower limb vascular blood flow surgery appears later than after one month, in our study this effect remained for 6 months. However, as mentioned above, our results showed that the new SEP and non-SET at home had significant effects on walking distance (total 6 MWT and PFW during 6 MWT), compared with the control group. A number of studies similarly reported that exercise training improved walking [23,24,25] and increased PFW distance in patients with PAD [26]. However, in these studies [23,24,25,26,27] patients were subjected to endovascular procedures, *i.e.*, percutaneous transluminal angioplasty (PTA), and the studied contingent consisted only of intermittent claudication patients. Only two randomized trials have described effects of exercise training after lower-limb bypass surgery [12,13]. Badger *et al.* showed that walking distance significantly improved in patients undergoing arterial bypass surgery after SEP. The mean increase in maximum walking distance was 3.8% in the control group (with standard preoperative and postoperative care) and 175.4% in the intervention group (with a SEP of twice-weekly treadmill assessments from 4 to 10 weeks postoperatively) [12]. Similarly, Lundgren *et al.* determined that the results of symptom-free walking distance (SFWD) and maximal walking distance (MWD) after reconstruction combined with physical training of patients with intermittent claudication are higher than in the following groups: reconstruction without physical training and physical training without reconstruction (SFWD—*p* *=* 0.0001, MWD—*p* *=* 0.0001). The patients were re-examined after at least 6 months of treatment. The training program comprised dynamic leg exercises three sessions per week, also patients were encouraged to perform the exercises during their leisure-time [13]. Some studies [9,10] evaluated the effectiveness of home-based exercise in patients with PAD; however, they included only patients with intermittent claudication who underwent pharmaceutical treatment but were not subjected to interventional therapy. Therefore, we were not able to compare these results due to the significant difference between the studied in relations to functional status. Considering the dynamics of walking distance after 6 months, the results of our investigation were worse than in the studies performed by Badger *et al.* and Lundgren *et al.* Our results may have been influenced by different methods of research; the studies above assessed walking distance of the studied patients by treadmill, while in our study—6 MWT was used. We have chosen 6 MWT, since functional performance measures are more strongly associated with physical activity levels during daily life than the treadmill walking measures [18]. So far, we did not find any studies that analyzed the effectiveness of SEP in a rehabilitation centre following lower-limb bypass surgery.

There is little correlation between the ABI change and improvement in symptoms or functional performance after revascularization. However, the ABI may still continue to improve for several weeks or months after revascularization [20]. Our control group had a worse ABI when compared with the patients in the intervention group, after 1, and 6 months; however, it did not reveal statistically significant difference between the groups given that the ABI estimates a whole-limb perfusion and cannot distinguish between graft failure and progression of PAD in the native arteries [20].

This finding was found by Bo *et al.*, the ABI results were not statistically significantly different between the intervention (12 weeks of SET) and control groups (without SET) during the 12 months of follow-up after PTA [26]. Also, Lundgren *et al.* did not determine significant difference in ABI between the group of reconstruction combined with physical training and the group of reconstruction without physical training [13]. However, Badger *et al.* reported an improvement in ABI with regular exercise in patients undergoing arterial bypass surgery after SEP [12]. As well as other researchers, we did not find significant differences perhaps due to the fact that ABI was assessed during rest while Badger *et al.* performed the assessment of preexercise and postexercise ABI [12]. Also, ABI results depend on age, which is as a result of arterial stiffening [20]. Badger *et al.* mean of age I group 72.5 ± 10.2 and II group 69.9 ± 9.5 [12]; in our and other studies mean age did not exceed 68 years.

QoL is reduced in older patients with PAD. Impaired walking results affected an ability to meet the personal, social, and occupational demands of daily living, thus it is important to assess patients’ perception of their QoL. The SF-36 questionnaire provides useful information about the impact of PAD on the patient’s life. Our results showed that the intervention group had significantly better results in physical and mental health component of QoL, compared with the control group. Similarly, Bo *et al.* obtained better results of the SF-36 in physical domain (*p* = 0.004) in the intervention group (hospital-based SET) than in the control group (standard care without SET) during the 12 months of the study after PTA [26]. However, Badger *et al.* report that surgery alone, as well as the exercise program, enhances QoL [12]. Similarly, improvement in SF-36 reported by Mazari *et al.* showed that there was no significant QoL advantage comparing PTA, SEP, and PTA plus SEP [27].

Some standard training methods are recommended for patients with PAD: a training at constant intensity and moderate speed during a set time period [28], and a training at a higher intensity with walking exercises maintained until discomfort or the onset of claudication stops the exercise [29]. Most studies used the above-mentioned methods for patients after endovascular treatment, except one study, which applied aerobic interval training [26]. This aerobic interval training program has been widely used in Scandinavian hospitals for patients with coronary artery disease. It is designed to improve physical capacity, body awareness, and emotional well-being, to promote a return to work and to improve prognosis [30]. To our knowledge, there are no other clinical studies that would compare aerobic interval treatment for PAD patients with commonly recommended exercise training methods after endovascular treatment or bypass surgery.

Our new PTP tasks were as follows: (1) to reduce limb symptoms because even postoperatively many patients still have claudication and limited activity due to decreased confidence and muscle wasting [12]; (2) to improve physical capacity, prevent or diminish physical disability, since PAD effects the function of locomotion resulting in disability [3,31]; (3) to reduce the frequency of cardiovascular cases, since PAD patients have higher risk to die of CVD than those who do not have this complication [6].

For the patients of the intervention group, the individual programs designed for patients with PAD as well as the program at home considering patient’s physical capacity were developed at the rehabilitation centers. These programs ensured a better and statistically significant patients’physical capacity, hemodynamic parameters and significantly improved quality of life indices in physical and mental domains. It suggests that the programs had been developed in accordance with the patients’ condition. Meanwhile, the control patients were performing rehabilitation procedures according to the general CVD program, which is designed for patients with cardiological diseases. Patients after lower-limb bypass surgery have been experiencing the pain in the lower extremities for a few weeks, thus their exercise tolerance differs from that in the patients who underwent coronary artery bypass surgery. Although both groups had SEP in the rehabilitation centers, specific activities were targeted at underlying and concomitant diseases that resulted in better results compared with the control patients. Acquired skills of physical activity in the rehabilitation center helps patients to gain confidence to continue exercises at home. Patients in the intervention group were more motivated to continue physical activity at home, since they were provided with recommendations and programs designed for the individual, while control patients received only recommendations to carry on physical exercises. All these factors led to better and statistically significant results in the intervention group.

There are a few limitations of the present study. Only patients with femoral-popliteal arterial disease were included, thus a large number of patients with bilateral or mixed arterial disease were excluded. However, this was necessary from a scientific point of view to reduce the influence of confounders, and to provide a robust answer to the treatment controversy in this group. Since the individual programs for the intervention group patients had been developed, volume of exercise was not controlled; thus programs of different physical load were applied. The results of our study were affected by other measures applied in the rehabilitation center, such as occupational therapy, physiotherapy, therapeutic massage, psychologist and social worker consultations.

## 5. Conclusions

In addition, patients who continue the new SEP and complete non-SET at home for 5 months have a significantly better functional status, greater scores in the physical health and mental health of QoL than those who continue the commonly used rehabilitation program. Thus, the systemic use of SEP for PAD patients starting from inpatient, rehabilitation centers and continuing the program at home provides a better biosocial functional restoration for rehabilitees. To improve the effectiveness of the treatment and rehabilitation, the new SEP is recommended for patients recovering from artery bypass grafting in rehabilitation centers with the further development of individual exercise training programs.
